# Multi-Omics Integration Reveals a Competitive Endogenous RNAs Network for the Identification of Progression Biomarkers and the Stratification of Patients Diagnosed With Nephroblastoma

**DOI:** 10.3389/fonc.2020.00444

**Published:** 2020-04-07

**Authors:** Jingbo Wang, Yuan Wang, Liang Han, Mohamed Shahen, Chaofeng Hu, Furong Li

**Affiliations:** ^1^Translational Medicine Collaborative Innovation Center, The Second Clinical Medical College (Shenzhen People's Hospital), Jinan University, Shenzhen, China; ^2^Integrated Chinese and Western Medicine Postdoctoral Research Station, Jinan University, Guangzhou, China; ^3^School of Nursing, Weinan Vocational and Technical College, Weinan, China; ^4^Department of Andrology, Fangshan Hospital, Beijing University of Chinese Medicine, Beijing, China; ^5^Zoology Department, Faculty of Science, Tanta University, Tanta, Egypt

**Keywords:** nephroblastoma, competitive endogenous RNA, tumor progression, prognostic marker, tumor categories

## Abstract

Specific types of nephroblastoma (Wilms' tumor, WT) are known to associate with poor overall survival. Emerging experimental evidence has demonstrated that competitive endogenous RNA (ceRNA) networks have important roles in regulating cancer occurrence, but the roles of ceRNAs in regulating the WT progression and the patient outcomes remain unclear. Using the multi-omics data of 132 WT patients collected from TARGET database, an integration analysis pipeline was performed to construct a highly reliable ceRNA network. As results, a total of 147 nodes (116 mRNAs, 15 miRNAs, and 16 lncRNAs) were identified and used to explore the underlying mechanism for WT progression. WGCNA analysis further identified several prognostic molecules, including hsa-mir-93, LINC00087 and RP5-1086K13, that significantly associated with the overall survival rate. And, enrichment analysis verified the participation of these molecules in tumor-related pathways, such as those controlling autophagy and cadherin-mediated adhesion. Importantly, the WT patients were classified into three categories according to the ceRNA network, which significantly correlated with the overall survival. In conclusion, the ceRNA network could be a promising tool to further validate the prognostic biomarkers and categories of patients diagnosed with WT.

## Introduction

Nephroblastoma, also known as Wilms' tumor (WT), is a complex childhood tumor of the kidney and the most prevalent type of kidney cancer in children (1). It is the underlying cause of 6–14% of children with tumors and up to 95% of all kidney cancers in children, affecting approximately one child per 10,000 worldwide under 15 years of age (2). Multiple effective treatments have been developed, and the overall survival rate of WT has reached 90% within the past several decades (3). However, recurrence still occurs in ~15% of WT patients with favorable pathological findings and practically 50% of anaplastic WT patients. Relapsed patients, as well as those with bilateral or unilateral high-risk tumors, who are at great risk for significant late effects of therapy, continue to have poor event free survival rates (4–6). Therefore, to develop novel and effective therapeutic approaches, it is important to understand the eventual downturn in the disease state that many WT patients will undergo.

Carcinogenesis is a complex process driven by abnormal gene combinations which may vary greatly between patients. WT is a genetically heterogeneous disease. Several gene alterations in predisposing loci have been well-identified, including mutations in Wilms' tumor gene 1 (WT1), catenin beta 1 (CTNNB1), insulin-like growth factor-2 (IGF2) and Wilms' tumor gene on the X chromosome (WTX) (7–9). The multifaceted regulation across multiple genes is important for the development of this disease. Numerous studies have reported that non-coding RNAs, including microRNAs (miRNAs) and long non-coding RNAs (lncRNAs), may play a critical role in cancer development (10, 11). Approximately 15% of patients diagnosed with WT have mutations in the miRNA-processing genes (12). Gong et al. analyzed the miRNA expression profiles of WT patients and identified 5 potential prognosis biomarker (13). Another study using microarrays to perform miRNA and gene expression profiles of WT patients and found the relationship between abnormally expressed miRNA and tumor progress (14). Previous studies have focus on the single omics and research on transcriptome has been largely limited to mRNA. As previously discussed, a single factor cannot explain the development of cancer. However, computational analysis provides a good way to understand the interactions between different factors that may contribute to cancer development.

Recent studies have demonstrated another layer of miRNA-mediated regulation that involves direct interactions between RNA molecules and common miRNAs. These RNAs, which were first presented in 2011 by Rubio-Somoza et al. (15), are known as competing endogenous RNAs (ceRNAs). The ceRNA hypothesis states that the pool of mRNAs, lncRNAs and other ncRNAs shares common microRNA response elements (MREs) with miRNAs, which can inhibit normal miRNA functions through competitive binding, thereby participating in the regulation of cell behavior (16). A recent bioinformatics study revealed that AFA-P1-AS1 acts as a ceRNA, competitively binding with miR-423-5p and directly regulating genes in the Rho/Rac pathway, thereby enhancing nasopharyngeal carcinoma cell migration and invasion (17). Another study characterized a ceRNA network that could distinguish the mesenchymal subtype from other glioblastoma subtypes (18). Taken collectively, these findings support the usefulness of the ceRNA network in understanding the development of this disease.

Most recent studies on the ceRNA network consider miRNAs to be gene regulators that act alone, and they ignore the influence of other regulatory factors on the ceRNA network, such as epigenetic factors, transcription factors and gene copy number variation factors (19). Losing sight of these critical factors may lead to spurious miRNA–gene interactions, which may cause false positive results in the ceRNA network. The recently proposed Cancerin algorithm overcomes this problem by integrating muti-omic data (20). Another problem, however, involves the identification and characterization of suitable candidate nodes to expand the ceRNA network. Most studies have used differential expression patterns as a screening standard. However, due to the inherent characteristics of transcriptomic data, there is usually severe noise in the differentially expressed genes (21). In addition, the genes with the greatest variations are not necessarily the genes responsible for the phenotypic changes because of the complex hierarchical relationships within the biological regulatory network. Therefore, it is critical to identify the transcripts most associated with cancer progression and to define them as nodes in the expansion of the ceRNA network. Weighted Gene Co-expression Network Analysis (WGCNA) solves this problem well (22). This method has been widely used to screen the gene co-expression module and the key node, collectively known as the hub node, which is most closely related to phenotypic changes.

A fundamental approach to study the heterogeneity of WT is to stratify the patients according to the molecular characteristics. Thus, tumors can be divided into clinically and biologically meaningful subtypes (23, 24), with each subtype associating with similar molecular markers. Previously, many attempts have been made to stratify various cancers using high quality transcriptomic signatures, and some molecular subtypes in breast cancer have now been clinically validated such as Mammaprint and Oncotype Dx (25). Since the endogenous RNA network integrates multiple layers of information, we hypothesized that the classification of patients using the ceRNA network would be useful.

In this study, we obtained RNA-seq, copy number and methylation data from the TARGET Database of 132 WT patients at different stages of the disease. The candidate miRNAs, mRNAs and lncRNAs related to tumor progression were identified by co-expression analysis. Furthermore, multi-omics data (genome, transcriptome, and epigenome) were integrated, and different methods were adopted to build a high-confidence ceRNA network. As a result, several key lncRNAs, which could predict patient prognosis, were identified and further investigated. According to these results, six lncRNAs could be used as reliable indicators of patient prognosis. In addition, we stratified patients using the condense cluster method and divided them into subtypes with significant clinical significance based on the ceRNA network (Figure S1). In summary, the ceRNA network obtained from this multi-group study represents an effective approach to study the stratification of patients, as well as to identify the mechanism responsible for the progression of WT.

## Materials and Methods

### Patients and TARGET Data Retrieval

The clinical data of 132 WT patients were obtained from TARGET (Therapeutically Applicable Research To Generate Effective Treatments) database. The survival and stage information were included in this database. The RNA-seq, miRNA-seq, DNA methylation and DNA copy number information were also downloaded. Patients with incomplete clinical information were filtered out, and 132 WT patients were retained. The study is in accordance with publication guidelines provided by TARGET (https://ocg.cancer.gov/programs/target/target-publication-guidelines). Since the data comes from the TARGET database, no further approval was required from the Ethics Committee.

### RNA Sequence Data Processing

The RNA FPKM (fragments per kilobase of exon per million fragments mapped) and miRNA expression data of 128 WT patients were obtained from TARGET database. All data from the samples were derived from the Illumina Hi-Seq platform and freely available to download. Furthermore, “annotable” package based on the R environment was used to distinguish the lncRNA and coding RNA. Finally, 20,347 mRNA, 5983 lncRNA and 1870 miRNA were identified.

### Copy Number Alteration

Mean copy numbers of chromosomal segments in the whole genome were provided by level 3 copy number alteration data from TARGET. Using the genomic location information of protein coding genes provided by GENCODE Release 26 (GRCh37), the R Bioconductor package CNTools were applied to transform the segmented CNA data into a gene-level data matrix where each entry represented copy number value of a gene in a definite sample.

### Methylation Data Pretreatment

The genome-wide methylation level of ~450,000 CpG sites needed to be measured in level 3 DNA methylation data from TARGET samples. The ratio of methylated probe intensity of the overall intensity (sum of methylated and unmethylated probe intensities) was defined as the methylation level of each CpG site (i.e., β value). Thus, β ranges between 0 and 1, with 0 being hypomethylated and 1 being hypermethylated. Previous research indicated that the methylation of CpG sites in the promoter regions resulted in gene expression change. Therefore, only considered β values of CpG sites are in genes' promoter regions. Thus, Bioconductor annotation package AnnotationHub was used to identify the probes positioned at the upstream 200–1,500 base pairs from the gene transcription start site. Gene's methylation level was estimated as the mean of its associated upstream probes' β values.

### Identification of Differentially Expressed Genes, miRNAs and lncRNAs

To identify mRNA, lncRNA, and miRNA which was associated with tumor progression, we divided the tumor samples into 2 groups (early stage and advanced stage) and used LIMMA package (R version 3.4.1) to analyze differences in the expression levels between the two tumor groups. As the raw transcriptomic data may be noisy, several filtering processes were performed to improve the quality of the expression profile. Firstly, we removed RNAs with low-expression values in more than 70% of the WT patients. Then, we calculated the coefficient of variation (CV) in gene expression for each RNA, and remove the 20% of RNAs with the lowest CV values. In addition, for multiple ensemble gene ids corresponded to the same gene symbol, the genes with maximum CV was retained to represent that gene. The differentially expressed mRNAs, lncRNAs and miRNAs were identified based on the same thresholds (absolute log 2 FC > 2.0 or *p* < 0.05).

For immunologic gene sets, we first captured relevant microarray datasets published in the immunology literature that has raw data deposited to Gene Expression Omnibus (GEO) with accession number GSE37301, GSE37605, GSE6259, and GSE2405. These studies included both human and mice data. However, it is proved that the characteristics of the activation of lymphocytes and differentiation of bone marrow cells were highly conserved between human and mouse cells (26). More importantly, instead of focusing on the changes of individual genes, we used the enrichment analysis to determine the overall coincidence degree of WT deterioration related genes and immune-related gene sets, which helped us identify the biological significance of different genes. For each published study, the relevant comparisons were identified (e.g., WT vs. KO; pre- vs. post-treatment etc.) and brief, biologically meaningful descriptions were created. All data were processed and normalized the same way to identify the gene sets, which correspond to the top or bottom genes (FDR < 0.02 or maximum of 200 genes) ranked by mutual information for each assigned comparison.

### Screening of Candidate Genes

Based on the previously identified mRNA, lncRNA, and miRNA, WGCNA was carried out to acquire candidate mRNA, lncRNA, and miRNA which is relative to clinical stages of the disease. The correlation of gene expression profile with module eigengenes (Mes) was defined as the module membership (MM) and the correlation between gene and external traits was defined as gene significance (GS) measure. The genes with |GS+ MM| ranked above the top 10% were selected as candidate gene together with genes in the core module.

### Functional Enrichment Analysis of Core Module Gene

For a deeper understanding of the biological effects and pathways of the aberrantly expression core module gene, Gene Ontology (GO) Biological Process, Kyoto Encyclopedia of Genes, and Genomes (KEGG) pathway analyses were constructed using the R/Bioconductor package of Clusteprofiler. Functional enrichment analysis was based on the threshold of *P* < 0.05. The predicted function of lncRNA is based on the function of the gene with the highest correlation with lncRNA in ceRNA network.

### Construction of ceRNA Network

In order to get a reliable ceRNA network, the candidate mRNA, miRNA and lncRNA which was selected in part 6 was invoked as nodes. Cancerin and GDCRNATools were utilized to construct the tumor deterioration-related ceRNA network. Cancerin integrated multidimensional cancer genomics data in order to infer cancer-associated ceRNA interaction networks which could identify the miRNAs contributed to the differential expression of RNA's. The newly developed GDCRNATools is used for deciphering the lncRNA-mRNA related ceRNA regulatory network as well as many routine analysis including functional enrichment analysis, DEG analysis and survival analysis in cancers. The code provided by Cancerin was used to integrate transcriptome, DNA methylation and copy number information to predict ceRNA network. Furthermore, ceRNA network was also predicted with GDCRNATools. Finally, union of the two ceRNA networks was used as the ceRNA network.

### Patient Stratification

In order to affirm patient stratification based on ceRNA network, a patient similarity matrix was constructed. Each element in the matrix represents the correlation coefficient based on ceRNA network. Then, the ConsensusClusterPlus package was used to reclassify patients. Furthermore, the similarity between samples was calculated by Pearson's correlation. Samples were distributed in k clusters by the PAM algorithm. The best number of clusters was determined by relative change in area under the CDF (Consensus Cumulative Distribution Function) curve compared k and k-1 (27).

### Survival Analysis

Survival analysis for all RNAs in the ceRNA network was carried out by using the R survival package (https://CRAN.R-project.org/package=survival, Version: 2.41-3). The log-rank test was carried out to identify whether the expression of lncRNAs, mRNAs and miRNAs was correlated with overall survival. For the overall survival rates, we use the log-rank test to compare the significant differences in univariate analysis between each subgroup. Unless otherwise specified, a *P* < 0.05 is considered as statistically significant.

### Data Availability

The datasets analyzed during the current study are available in the TARGET repository (https://ocg.cancer.gov/programs/target/data-matrix). All relevant data are within the paper and its Supporting Information files.

## Results

### Gene Expression Patterns in WT Patients at Different Stages of the Disease

**Figure 1:**
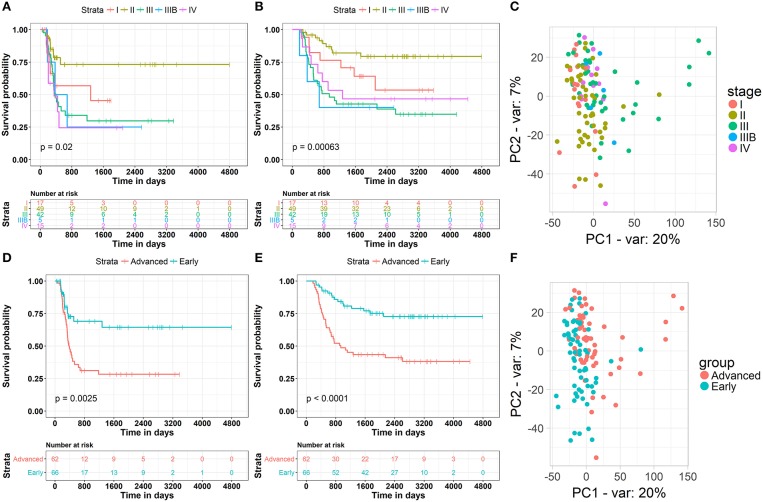
Clinical and gene expression patterns of WT patients at different stages of the disease. **(A)** Kaplan-Meier curve analysis of the Disease-Free survival rate in WT patients at different stages. **(B)** Kaplan-Meier curve analysis of the overall survival rate in WT patients at different stages. **(C)** Principle-component analysis of RNA sequencing (RNA-seq) of WT patients at different stages, percentage of variance (% of var) indicated. **(D)** Kaplan-Meier curve analysis of the Disease-Free survival rate in WT patients in different groups. **(E)** Kaplan-Meier curve analysis of the overall survival rate in WT patients in different groups. **(F)** Principle-component analysis of RNA sequencing (RNA-seq) of WT patients in different groups, percentage of variance (% of var) indicated.

The clinical data of 132 WT patients at different stages of the disease were downloaded from the TARGET Database, including progression-free survival, total survival, disease stage, and the corresponding RNA-seq data. The relationship between patient survival and disease stage, which was first examined in our study, indicated that the traditional stage significantly correlated with the patient disease-free survival and overall survival rates (Figures 1A,B). Surprisingly, the worst disease-free survival rate was not observed in patients at the latest stage of the disease. For example, we found by disease-free survival analysis that patients at stage II were better than those at stage I, and similar results were observed by overall survival analysis. The most likely cause of this result is that there are a few samples of this tumor and fewer clinical samples have been collected, resulting in some deviations in survival statistics. Another possibility is that due to the difficulty in early diagnosis of cancer, some patients were found in stage I, and in fact the tumor development was very close to stage II, and even worse than the normal stage II patients in terms of partial molecular expression. Principal component analysis (PCA) was used to reduce the dimensionality of the gene expression data and to visualize two components on the scatter plot. The gene expression data of patients at different stages of the disease did not significantly cluster (Figure 1C). This may have been due to the fact that conventional disease stages tend to focus on phenotypes, such as tumor size and metastasis, while ignoring genotypes and molecular mechanisms, which may underlie ineffective treatments. Considering the contradiction between the survival rate of patients and the early stages of the disease, and the difficulty in discerning the survival rate and the late stages of the disease, we first classified phase I and II patients as “early,” whereas the remaining patients were classified as “advanced” to obtain information on the key biomolecules involved in cancer progression. According to results from survival analysis, “early' patients were far superior to “advanced” patients (Figures 1D,E) in both the disease-free progression survival rate and the total survival rate. More importantly, PCA analysis showed that patients at an “early” stage presented different patterns of gene expression than those at an “advanced” stage (Figure 1F), suggesting that several key molecules are involved in the progression of renal myoblastoma. These results lay the foundation for the search of biomarkers associated with progression.

### Clinical Relevant Candidate Nodes Identification

To identify candidate molecules associating with the eventual downturn and poor prognosis of nephroblastoma, WGCNA was used to analyze the co-expression network of mRNA-miRNA-lncRNA. First of all, a differentially expressed gene was defined as a gene whose log2 value of the fold change of the expression (logFC) was >2 and the *P* < 0.05. We identified 4,285 differentially expressed mRNAs and 246 differentially expressed lncRNAs using the LIMMA R Package. The differentially expressed RNAs are listed in Table S1. We used a similar procedure and identical criteria to screen the differentially expressed miRNAs and identified 259 differentially expressed miRNAs (Table S2).

**Figure 2:**
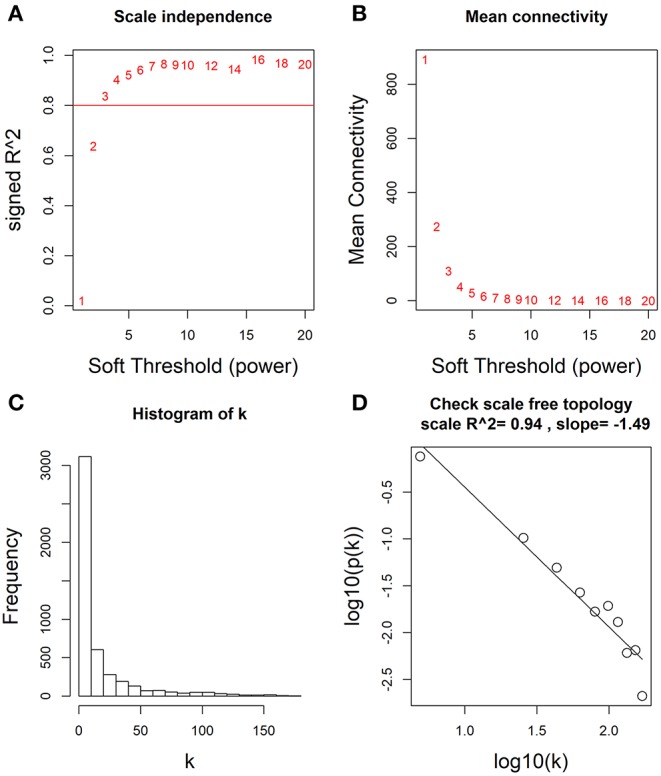
Determination of soft-thresholding power by WGCNA. **(A)** Analysis of the scale-free fit (i.e., *p(k)* ~ *k*^−γ^) index for various soft-thresholding powers (β). **(B)** Analysis of the mean connectivity for various soft-thresholding powers. **(C)** Histogram of connectivity distribution when β = 4. **(D)** Checking the scale free topology when β = 4.

Based on these differentially expressed mRNAs, miRNAs and lncRNAs, we analyzed their co-expression networks using WGCNA to identify the most relevant molecular modules associating with clinical decline. Soft-threshold beta was selected as a suitable weighted parameter of the adjacency function before constructing the weighted co-expression network. After performing the calculation, we selected the correlation coefficient closest to 0.8 (soft-threshold catcher = 4) to construct the gene modules using WGCNA (Figures 2A,B). After determining the soft threshold, all differentially expressed molecules (mRNAs, miRNAs and lncRNAs) were used to construct the weighted gene co-expression network. We found that the degree of node conformed to the power law distribution (Figures 2C,D).

**Figure 3:**
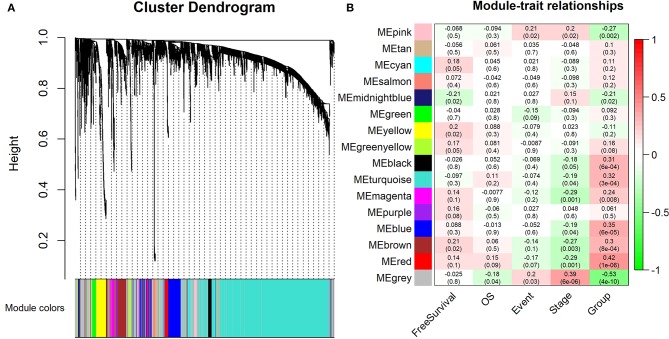
Identification of the modules associating with the clinical traits of WT patients. **(A)** Dendrogram of all differentially expressed RNAs clustered based on a dissimilarity measure (1-TOM). **(B)** Heatmap of the correlation between module eigengenes and clinical traits of WT patients. “Event” is binary value indicating the death statue of each patient (0 for alive and 1 for dead), and “Group” represents the progression status of each patient (0 for early and 1 for advance).

Furthermore, 15 modules were identified through business linkage hierarchical clustering (Figure 3A). The red module was found to have the highest association with tumor progression (*R*^2^ = 0.42, *P* < 0.01) (Figure 3B), and this module was selected as a clinically significant gene set for further analysis. To avoid missing other key genes related to tumor progression, highly expressed molecules of module membership (MM) and gene significance (GS) were added to the candidate node collection. We selected mRNAs, miRNAs and lncRNAs with cut-off criteria (|MM+GS| ranked above top 10%). After integrating the molecules in the previously established core modules, we obtained 458 mNRAs, 26 miNRAs, and 33 lncNRAs as the final molecule nodes to build the ceRNA network. The complete list of candidate molecules is presented in Table S3.

**Figure 4:**
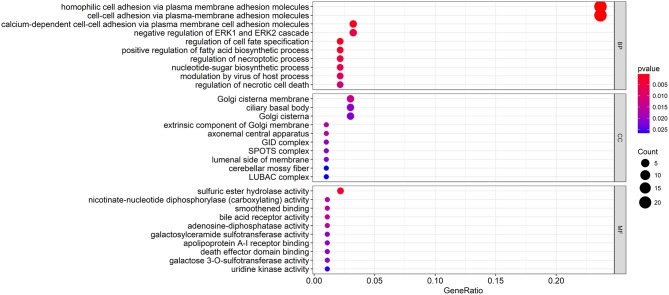
Functional enrichment analysis of the candidate genes. The GO terms with the most significant *p*-values. The x-axis represents the number or gene ratio of core module mRNAs involved in the enrichment terms.

To explore the biological relevance between the candidate genes and clinical decline, Gene Ontology (GO) function and pathway enrichment analyses were performed using the R clusterProfiler Package (28) (Table S4). The results showed that the functions of the candidate genes mainly concentrated in areas of cell adhesion and cell fate specification (Figure 4A), which may ultimately contribute to cancer progression. As the immune regulation controls clinical development, we obtain immunologic gene sets from microarray gene expression data from immunologic studies and investigate whether our candidate genes are related to the innate immune system (Figure S2). In addition, we found that many immune-based mechanisms were related to the poor prognosis of WT, including the up-regulation of B cell–T cell interactions and foxp3 fusion (Figure S3).

### Construction of High Confidence ceRNA Competition Network

**Figure 5:**
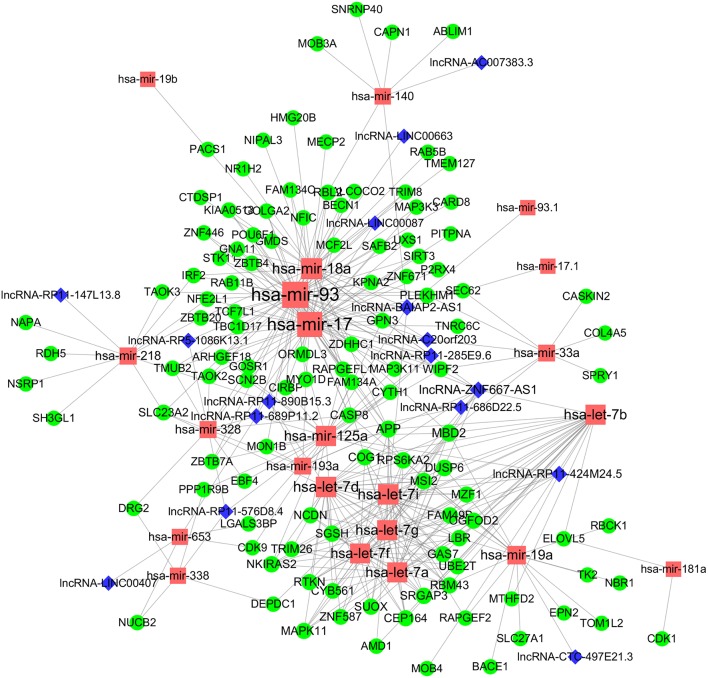
CeRNA regulatory network in WT. The nodes highlighted in red indicate candidate miRNAs, the nodes highlighted in blue indicate candidate lncRNAs, and the nodes highlighted in green indicate candidate mRNAs. The size of the point represents the connectivity of the node, that is, the number of other points connected. Edge represents an interaction between two nodes.

**Figure 6:**
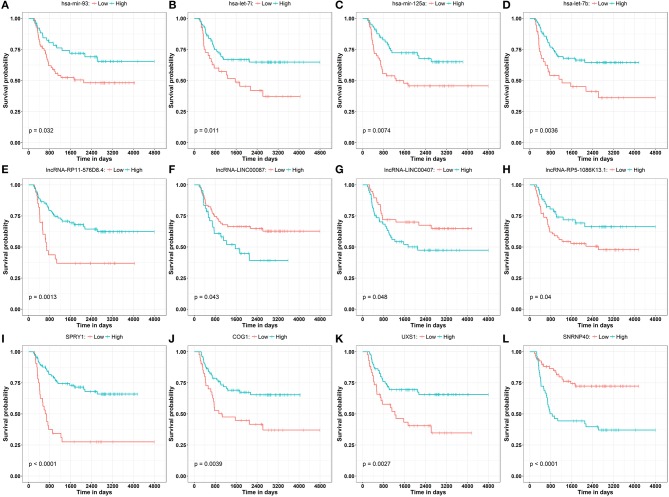
Kaplan–Meier curve analysis of the disease progression involving miRNAs **(A–D)** has-mir-93, has-let-7i, has-mir-125a, has-let-7b, lncRNAs **(E–H)** lncRNA-RP11-576D8.4, lncRNA-LINC00087, lncRNA-LINC00407, lncRNA-RP5-1086K13.1, and mRNAs **(I–L)** SPRY1, COG1, UXS1, SNRNP40 in WT patients.

To obtain a comprehensive and highly reliable ceRNA competitive network, we combined the results of two newly published ceRNA network prediction methods, namely, Cancerin and GDCRNAtools. Cancerin incorporates multi-omics information from patients, including epigenetic, genomic and transcriptomic information, to increase the reliability of the network. In this study, miRNA–mRNA and miRNA–lncRNA relations with media confidence <0 were selected as the interactive relations of the ceRNA network. The parameter “media confidence” is derived from the Cancerin to filter the high reliable miRNA–mRNA and miRNA–lncRNA associations. Briefly, Cancerin estimated the confidence intervals of the correlation coefficient between one miRNA and each of its targets using LASSO regression for 500 time, and the median of the mid-point points of the 500 confidence intervals was defined as media confidence. And, “media confidence <0” reflects reliable negative correlation relationship between the expression of a miRNA and its target. According to this method, 122 miRNA-target interactions and 64 nodes, including 39 mRNAs, 20 miNRAs and 5 lncRNAs, were selected (Table S5). In addition, we predicted 103 nodes and 330 edges in the ceRNA network using GDCRNA tools (Table S6). After combining these two sets of results, we constructed the endogenous competitive network of mRNA–miRNA–lncRNA (Figure 5, Table S7). This network contained 15 miRNAs, 16 lncRNAs, 116 mRNAs, and 407 interactions. The topological properties of the nodes in the network were further analyzed, and we found that hsa-miR-93 had the highest degree (degree = 58), which represents the number of targets it interacts with. We investigated the relationship between this miRNA and patient survival (Figure 6A) and confirmed the significant correlation between has-mir-93 and the overall survival (*p* < 0.05). In addition, we investigated the relationship between other nodes in the network and patient survival, and found that 39.46% of the nodes correlated with patient survival (*p* < 0.05, Table S3).

**Figure 7:**
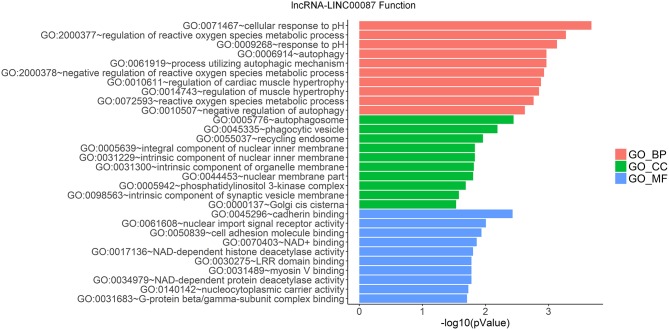
Gene ontology enrichment analysis of LINC00087-regulated genes. The x-axis represents the –log10 (*P*-value) of the enrichment analysis. Red, green and blue represent the enrichment of Gene Ontology (GO) Biological Process, Cellular component, and Molecular function, respectively.

**Figure 8:**
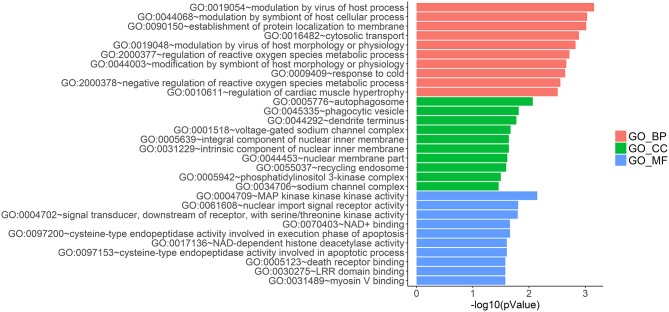
Gene ontology enrichment analysis of RP5-1086K13-regulated genes. The x-axis represents the –log10 (*P*-value) of the enrichment analysis. Red, green and blue represent the enrichment of Gene Ontology (GO) Biological Process, Cellular component and Molecular function, respectively.

LncRNAs regulate the expression of mRNAs through their interactions with miRNAs in the ceRNA network. Thus, the functions of lncRNAs can be reflected through the mRNAs regulated by them. In this study, two prognostic lncRNAs, LINC00087 and RP5-1086K13, were chosen for enrichment validation. We conducted GO enrichment analysis (GO_BP: biological processes, GO_CC: cellular component and GO_MF: molecular function) of their regulated mRNAs, and found that LINC00087 was mainly involved in autophagy and cadherin binding (Figure 7). RP5-1086K13 was involved in autophagosome dynamics and MAPKKK activity (Figure 8).

### Patient Stratification Based on the Clinically Relevant ceRNA Network

**Figure 9:**
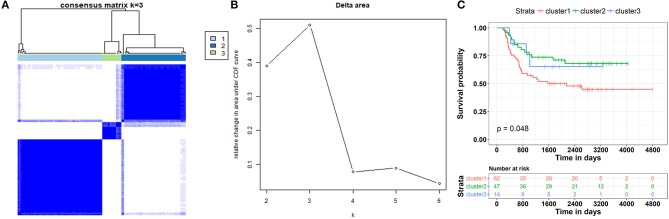
Patient stratification based on clinically relevant ceRNA network. **(A)** Consensus matrices represented as heatmaps for the chosen optimal cluster number (*k* = 3) for the WT patients. Patient samples are both rows and columns, and consensus values range from 0 to 1. The dendrogram above the heatmap illustrates the ordering of patient samples in 3 clusters. **(B)** Corresponding relative change in area under the cumulative distribution function (CDF) curves when cluster number changing from k to k + 1. The range of k changed from 2 to 10, and the optimal *k* = 3. **(C)** Kaplan-Meier curve analysis of the overall survival rate in WT patients at different clusters.

These results revealed that the ceRNA network was useful in the identification of the prognostic biomarkers for WT. They also implied a correlation between the ceRNA network and the patient's clinical prognosis. Therefore, the subtypes of the patients based on this ceRNA network were further investigated. We first extracted the expression information for each node involved in the ceRNA network of each patient. Partitioning around methods (PAM)-based consensus clustering, followed by cluster reliability analysis (Methods), was used to investigate the case of dividing patients into k (*k* = 2, 3, 4, 5, 6). Figure 9B shows the relative change in the area under the consensus cumulative distribution function (CDF) curve by comparing k with k-1. According to these results, we found that the patients were classified into three categories using optimal classification standards, and the patients presented a relatively obvious clustering pattern (Figures 9A,B). To confirm whether our classification had clinical significance, the relationship between the new staging and the total survival were calculated. We found that there was a significant correlation between the staging of patients based on the ceRNA network and the total survival (Figure 9C, *p* < 0.05).

## Discussion

The identification of genes and molecules that are related to tumor staging and progression is important for our understanding and control of the disease. WT is responsible for ~95% of kidney tumors in children. Although improved therapies and prognosis methods have greatly increased the survival rate, additional effort is still needed to deal with the disease since 50% of children who relapse go on to die (29). In this study, a multidimensional network was constructed to provide new insights on the mechanism underlying the progression of WT.

The relationship between the survival rate and the histopathological grades was the first consideration in our study, and the general trend is that higher grades were significantly associated with a lower overall survival rate. However, according to the result of PCA, no distinct RNA expression patterns were observed in different grades (Figure 1C), indicating that the histological grades in this cohort may not be sufficient to represent patients with subtypes for further deciphering the molecular mechanism of WT progression. Taking these contradictions into consideration, we divided WT patients into “early” and “advanced” stages, and identified the different gene expression patterns between the two stages (Figures 1D–F). Because the progression-free survival and overall survival rates of “early” patients were far superior to those of “advanced” patients, it is possible that key biomolecules exist to influence the clinical decline of patients diagnosed with WT. To confirm the candidate molecules related to this process, WGCNA was carried out to construct a weighted co-expression network (Figures 2A,B). Recent research by Meng et al. which employed WGCNA, identified major genes that mediate immune cell activation and mitosis (30). Another WGCNA study by Guo et al. disclosed key genes with aberrant expression levels that may participate in the pathogenesis of head and neck squamous cell carcinoma (31). WGCNA has also been used to identify potential prognostic markers for uveal melanoma, Glioblastoma Multiforme, lung cancer, bladder cancer and so on (32–35). While for WT, WGCNA analysis has been used in one research to identify hub genes associated with high-risk pathogenesis (36). These results indicate that this method can be used to identify new candidate nodes in the ceRNA network. After constructing this co-expression network, we found that it was a scale-free network, which conforms to conventional biological network features (Figures 2C,D). Furthermore, molecules with high MM and GS were considered as node molecules, as well as previously identified modules. The network, which was constructed based on all the nodes, played an important role in the understanding the mechanism underlying the clinical decline of WT patients.

To further understand the relationship between these candidate molecules and WT progression, functional enrichment analysis was performed, and the results indicated that the functions mainly concentrated in areas such as cell adhesion, the ERK pathway and necrosis (Figure 4). As previously described, cell adhesion plays a major role in tumor biology. Research carried out by Liu et al. showed that the high expression of the focal adhesion protein kindlin-2 in solid tumors, as a prognostic biomarker, may indicate poor outcome in patients (37). Cellular glycosylation, which participates in cell–cell recognition, communication and adhesion, has a major impact on the acquisition of malignant characteristics of gastric carcinoma (38). On the other hand, the cell surface CD56 glycoprotein, which controls cell adhesion and signaling, acts as a key biomarker in WT stem and progenitor cells (39). Another study demonstrated that ERK signaling may contribute to WT development (40). These results clearly support the reliability of our network analysis. Because interactions between the tumor and the immune system are also known to influence tumor progression, we analyzed the relationships between candidate genes and the immune system. We found that most genes were enriched in the innate immune system. We further explored the detailed immune-based functions, and found an up-regulation in Treg activity by our candidate genes (Figure 4C). An immunosuppressive microenvironment is essential for tumor progression, and Treg is an important supporter of this environment (41). Interestingly, a more pronounced Treg-induced cytokine response was observed in WT patients, according to previous studies. To reverse the Treg activation induced by the candidate biomolecules may prevent the clinical decline of WT patients.

In this study, Cancerin and GDCRNAtools were combined to construct a superior confidence ceRNA competitive network (Figure 5). Among all nodes in this network, hsa-miR-93 had the highest degree, and it was significantly related to the overall survival of patients (Figure 6A). Previous studies have already reported exosomal miR-93 to be a novel biomarker for both the diagnosis and prognosis of hepatocellular carcinoma and triple negative breast cancer (42, 43). However, there were few studies addressing its roles in WT patients. Our results indicated that hsa-miR-93 may be defined as a prognostic biomarker of this disease.

In the ceRNA network, the competitive binding of mRNAs and lncRNAs to miRNAs can regulate mRNA expression. Therefore, we carried out enrichment analysis of mRNAs regulated by lncRNAs to reflect the function of lncRNAs. LINC00087 and RP5-1086K13 were selected as prognostic biomarkers. LINC00087 is involved in autophagy and cadherin binding, whereas RP5-1086K13 is mainly involved in autophagosome dynamics and MAPKKK activity (Figures 7, 8). Autophagy is crucial for aggressive tumor growth, and this process is deregulated in WT patients (3, 44, 45). Cadherin belongs to a transmembrane superfamily of proteins, and E-cadherin is used for the diagnosis and prognosis of epithelial cancers (46). Decreased E-cadherin expression has been shown to correlate with a higher stage of WT (47, 48). On the other hand, the MAPK pathway is involved in numerous biological processes, including immunity, cell proliferation and tumor-related events, and it plays an important role in the progression of WT (49, 50). The participation of LINC00087 and RP5-1086K13 in these biological processes indicated that they have key roles in the progression of WT. However, further studies are needed to validate the precise mechanisms. The identification of prognostic biomarkers in the ceRNA network pointed to the potential relationship between the network and the clinical outcome of patients. We classified the patients into three categories based on this network (Figure 9). A remarkable correlation was observed between the staging of patients and survival.

Although we have integrated multiple omics data in this study and tried to construct a reliable and clinically significant ceRNA network for the study of prognosis and classification of WT patients, some limitations are still worth noting. First and foremost, as a rare tumor, WT has relatively scarce clinical samples, which lead to fewer omics data for analysis, which may to some extent improve the noise of omics data and reduce the reliability of the whole analysis. In addition, the scarcity of data will increase the difficulty of the found that clinical samples potential rules and patterns, and makes the extracting potentially meaningful and biologically relevant information more difficult. Finally, this research mainly focuses on the use of bioinformatics tools mining WT patients deterioration of underlying mechanisms and biomarker, the results remain to be further investigated *in vitro* and *in vivo*. There is reason to believe that in the near future, along with the accumulation of WT clinical and omics data, various experimental methods to further improve, partly as a result will be confirmed in this article, and may be directly used for clinical purposes. In summary, our results showed that the ceRNA network enhanced the molecular staging of cancer patients. Thus, it may facilitate the development of accurate treatment strategies for patients.

## Data Availability Statement

The datasets analyzed during the current study are available in the TARGET repository (https://ocg.cancer.gov/programs/target/data-matrix).

## Author Contributions

JW and FL: conceptualization and writing—review and editing. YW and LH: data curation. LH and MS: formal analysis. YW and CH: investigation. LH and CH: methodology. JW: project administration. MS: resources. FL: supervision. JW: writing—original draft.

### Conflict of Interest

The authors declare that the research was conducted in the absence of any commercial or financial relationships that could be construed as a potential conflict of interest.

## Supplementary Material

Figure S1 Analysis pipeline of the research.

Figure S2 The pathway terms (from MsigDB) with the most significant p-values.

Figure S3 The immunologic terms (from MsigDB) with the most significant *p*-values. The x-axis represents the number or gene ratio of core module mRNAs involved in the enrichment terms.

Table S1 The differentially expressed RNAs.

Table S2 Differentially expressed miRNAs.

Table S3 The complete list of candidate molecules of ceRNA network.

Table S4 GO function and pathway enrichment results of candidate genes.

Table S5 List of the selected target of miRNA.

Table S6 103 nodes and 330 edges of the ceRNA network.

Table S7 Intergrated ceRNA list.
