# Supplementary material for: Multi-Omics Integration Reveals a Competitive Endogenous RNAs Network for the Identification of Progression Biomarkers and the Stratification of Patients Diagnosed With Nephroblastoma
Source: Front Oncol. 2020 Apr 7;10:444. doi: 10.3389/fonc.2020.00444 (PMC7154083; doi:10.3389/fonc.2020.00444)
Supplement: Figure S1 — Analysis pipeline of the research. [file Data_Sheet_1.zip › supplementary figures.docx]

**Multi-omics integration reveals a competitive endogenous RNAs network for the identification of progression biomarkers and the stratification of patients diagnosed with nephroblastoma**

Jingbo Wang^1,2*^, Yuan Wang^3^, Liang Han^4^, Mohamed Shahen^5^, Chaofeng Hu^2^ and Furong Li^1,2*^

1. Translational Medicine Collaborative Innovation Center, The Second Clinical Medical College (Shenzhen People’s Hospital), Jinan University, Shenzhen, 518020, China.
2. Integrated Chinese and Western Medicine Postdoctoral research station, Jinan University, Guangzhou 510632, China.
3. School of Nursing, Weinan Vocational and Technical College， Weinan, Shaanxi 714026, China
4. Department of Andrology, Fangshan Hospital，Beijing University of Chinese Medicine, Beijing, 102400，China
5. Zoology department, faculty of science, Tanta University, Tanta 31527, Egypt

*Correspondence: [wangjingboqd2008@163.com](mailto:wangjingboqd2008@163.com) and [frli62@163.com](mailto:frli62@163.com)


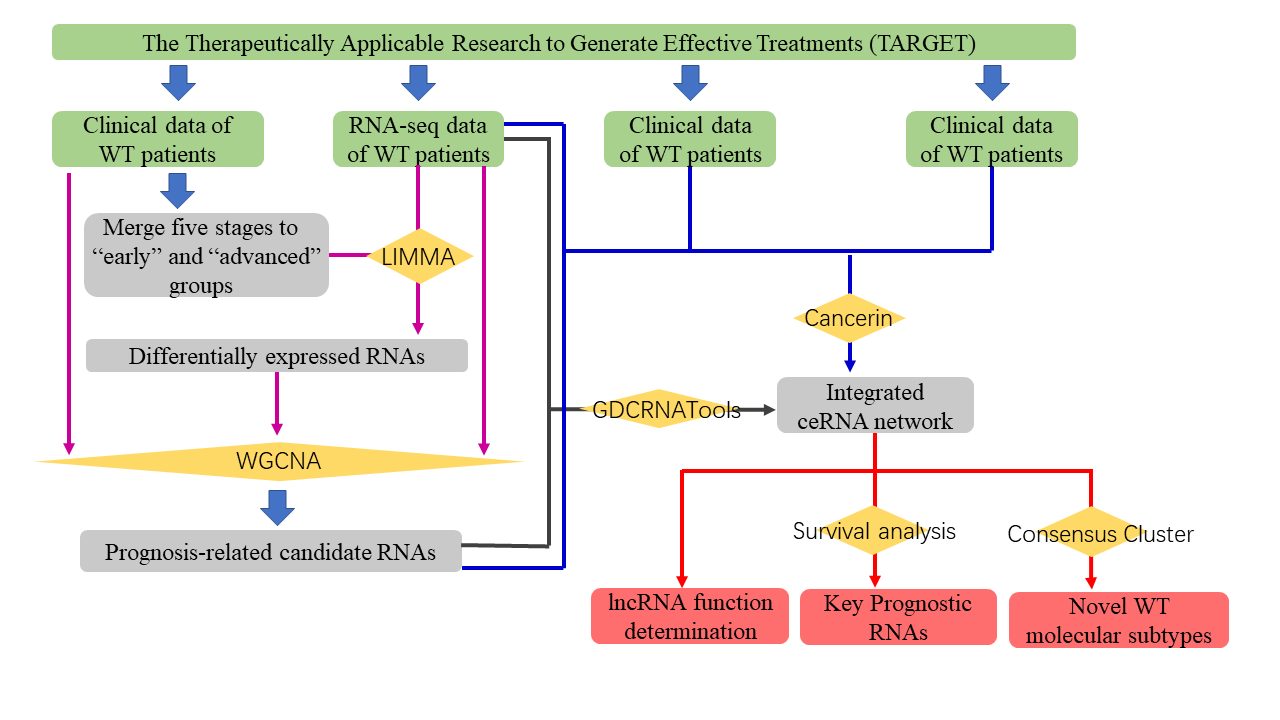


**Figure S1. Analysis pipeline of the research.**


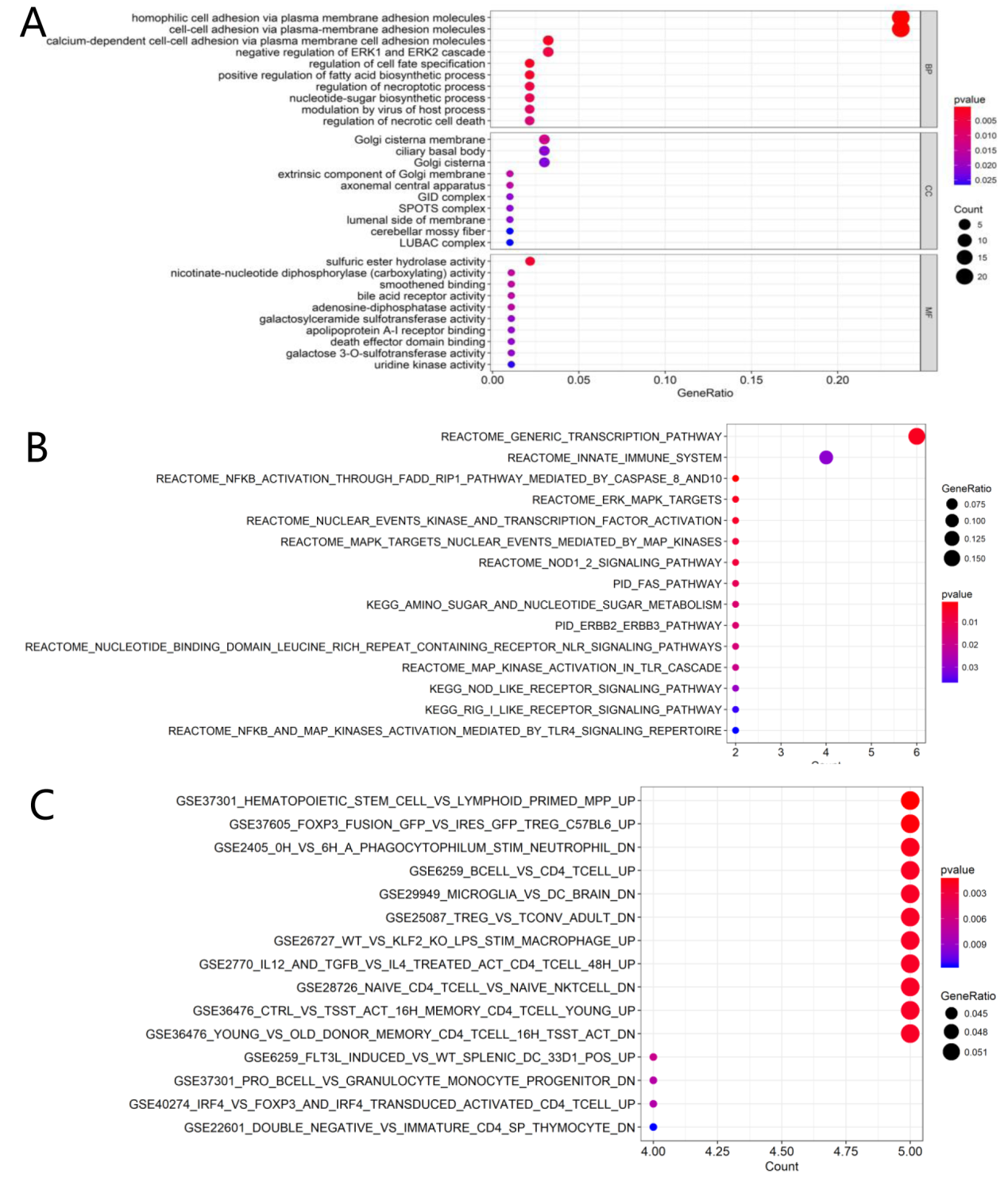


Figure S2. The pathway terms (from MsigDB) with the most significant p-values.


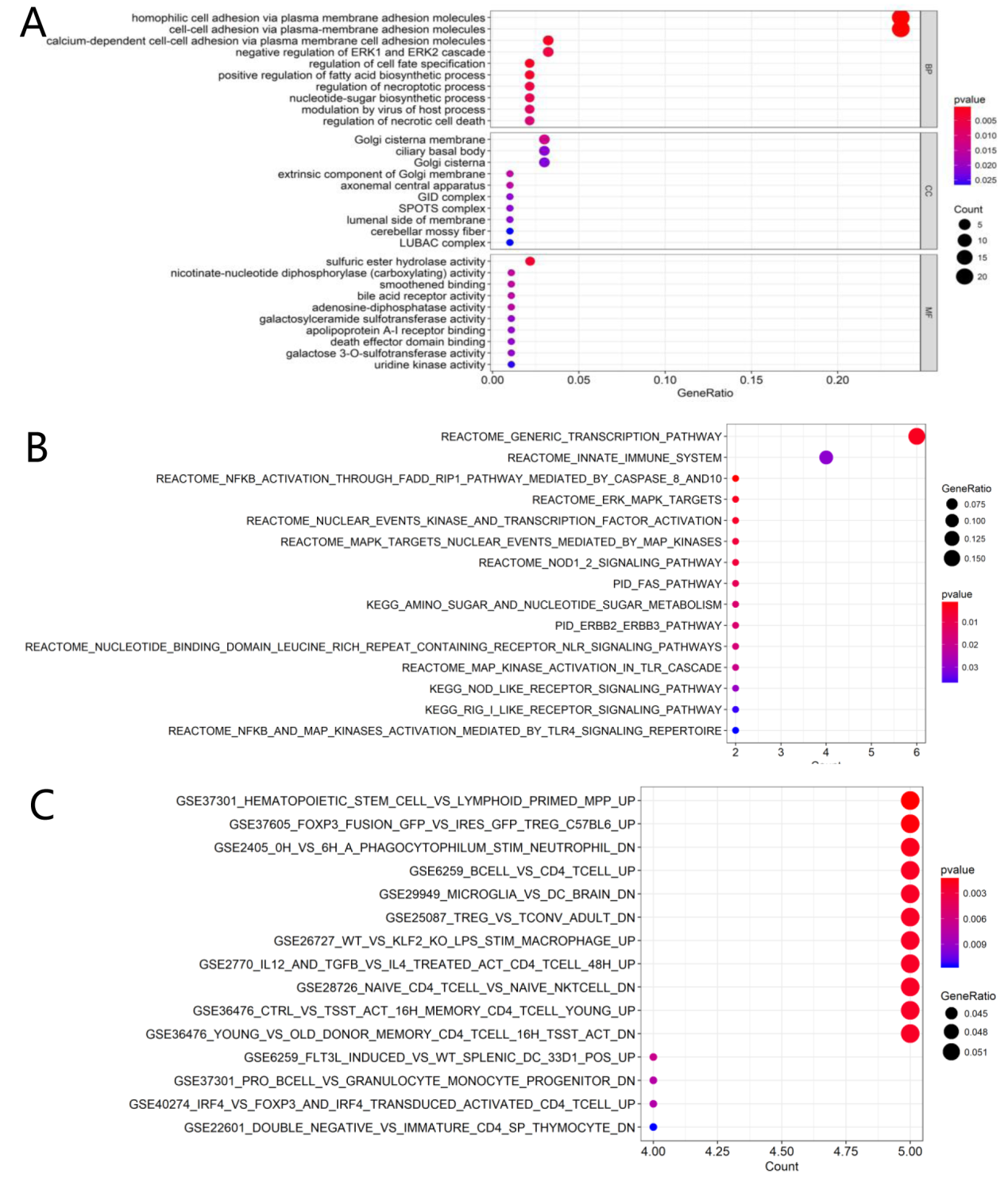


Figure S3. The immunologic terms (from MsigDB) with the most significant p-values. The x-axis represents the number or gene ratio of core module mRNAs involved in the enrichment terms.
